# Editorial: Acute phase proteins as biomarkers and therapeutics in acute and chronic inflammatory conditions

**DOI:** 10.3389/fphar.2023.1145990

**Published:** 2023-02-02

**Authors:** Frauke Stanke, Sabina Janciauskiene, Beata Olejnicka

**Affiliations:** ^1^ Department of Pediatric Pneumology, Allergology and Neonatology, Hannover Medical School, Hanover, Germany; ^2^ Biomedical Research in Endstage and Obstructive Lung Disease Hannover (BREATH), German Center for Lung Research, Hannover Medical School, Hanover, Germany; ^3^ Department of Respiratory Medicine, German Center for Lung Research, Hannover Medical School, Hanover, Germany

**Keywords:** muse cells, interleukin-1β, quercetin, inflammation, CRP-C-reactive protein, A1AT, alpha 1-antitrypsin, SLPI protein

This special issue includes two original articles and two reviews, which focus on anti-inflammatory properties of acute phase proteins (APPs), endogenous multilineage-differentiating stress enduring (Muse) cells, and exogenous flavonoid (quercetin).

The magnitude and rapidity of the change in the concentrations of acute phase proteins (APPs) play an important role in the establishment of host defense. APPs are implicated in many aspects of inflammatory and infectious diseases *via* eliminating pathogens, activating the complement system, neutralizing enzymes, scavenging free radicals, and modulating immune response among others. The expression of APPs is often abnormal in chronic disease states, however, changes in APPs may also vary among individuals due to genetic, nutritional, age, or other health-associated factors. Various pro- and anti-inflammatory properties ascribed to individual APPs make them important diagnostic biomarkers in health and diseases, and useful therapeutics for certain pathological conditions.

Richter and colleagues focused on three APPs, such as alpha-1-antitrypsin (AAT), C-reactive protein (CRP), and secretory leukocyte protease inhibitor (SLPI), as important regulators of IL-1β, the prototypical pro-inflammatory cytokine which contributes to the pathogenesis of several conditions, including cardiovascular diseases. Therefore, the mechanisms and agents controlling IL-1β expression and secretion represent a great interest in clinical and experimental research. The biosynthesis of pro-IL-1β is typically induced during the activation of pattern recognition receptors, such as Toll-like receptors (TLRs), by danger-associated or pathogen-associated molecular patterns (DAMPs or PAMPs) signaling *via* the transcription factors nuclear factor-κB or activator protein 1. While other cytokines are released upon biosynthesis, pro-IL-1β is not bioactive and stays within the cell unless a second danger signal induces the assembly of multi-protein complexes and inflammasomes. Extracellular adenosine 5′-triphosphate (ATP) typically acts as a second danger signal activating the ATP-sensitive P2X7 receptor, an ion channel, that induces the assembly of the NLRP3 (NACHT, LRR, and PYD domains-containing protein 3) inflammasome, activation of caspase-1, the cleavage of pro-IL-1 β and secretion of mature IL-1β. Authors of this review discuss how CRP, AAT, and SLPI can inhibit the ATP-induced NLRP3 inflammasome-dependent maturation and release of IL-1β, and provide evidence that the control of IL-1β release may involve the activation of unconventional nicotinic acetylcholine receptors which inhibit the ionotropic function of the ATP receptor P2X7. In fact, AAT is a member of serpin (serine protease inhibitor) superfamily and is a major inhibitor of neutrophil elastase and proteinase 3, but also expresses other anti-inflammatory properties, some of which are not related to protease inhibition. CRP belongs to the family of pentraxins and is regarded as an opsonizing agent that binds to bacteria and damaged host cells thereby contributes to their inactivation and elimination. Finally, SLPI is a whey acidic protein (WAP) family member constitutively produced and secreted by human epithelial cells. Independently of its anti-protease function, SLPI expresses anti-microbial, anti-inflammatory, and tolerogenic activities.

However, despite the diverse structural and functional properties, CRP, AAT, and SLPI seem to be important regulators of IL-1β levels.

Janciauskiene and colleagues used human peripheral blood mononuclear cell (PBMC)-based models with an aim to investigate further how AAT regulates lipopolysaccharide (LPS)-induced IL-1β release. Authors point out that the mechanisms regulating IL-1β secretion are dependent on the cell type. In contrast to murine macrophages and myelomonocytic cell lines, primary human PBMCs after triggering TLRs do not require a second signal for processing and secretion of IL-1β. Indeed, authors show that adding LPS to human PBMCs is enough to increase significantly *Il1B* mRNA, pro-IL-1β formation, and IL-1β release. By using this model, authors provide novel evidence that the regulation of IL-1β release by AAT may depend on the availability of free Cys232 in AAT protein. On the other hand, authors point out that a redox-dependent IL-1β secretion might also depend on the cell type. For example, the exposure to reducing agents and down-modulation of thioredoxin seem have opposite effects on the IL-1β secretion in human primary monocytes and in human leukemia monocytic cell line, THP-1. Since decades AAT protein is used as a therapy for emphysema patients with inherited AAT deficiency, therefore it is of clinical importance to investigate thoroughly biological properties of AAT.

Nowadays protein and cell-based therapies are used for various clinical applications. Kuroda and colleagues reviewed multilineage-differentiating stress enduring (Muse) cells, endogenous pluripotent-like reparative stem cells, which possess unique immunomodulatory characteristics, not seen in other stem cells. This review details the molecular and cellular properties of Muse cells as well as their capacities to repair tissues and tissue functionality, highlighting cell potential in regenerative medicine. The pluripotent Muse may be a next-generation cell therapy for inflammatory and tissue destructive diseases. As described in different animal models, Muse cells can selectively accumulate at the damage site by sensing the sphingosine-1-phosphate, a key mediator of inflammation. At the damage site, these cells can deliver anti-inflammatory, anti-apoptotic, anti-fibrotic, and tissue-protective effects, and repair the tissue by replacing damaged cells by differentiation into tissue-constituent cells. Muse cells have been detected in peripheral blood, specifically in stroke patients during the acute phase. A beneficial feature of donor-derived allogenic Muse cells is that these cells can be directly administered to patients without HLA matching or immunosuppressive therapy.

Quercetin is a natural flavanol antioxidant found in various fruits and vegetables that is known to act as anti-inflammatory, antiviral and anti-fibrosis agent, and is used for different therapeutic applications. Geng and colleagues investigated effects of quercetin on macrophage senescence and pulmonary fibrosis, and explored underlying mechanisms. Authors provide experimental evidence that quercetin inhibits macrophage senescence and reduces pulmonary fibrosis. Quercetin specifically seems to decrease the expressions of the senescence-associated secretory phenotype (SASP), including proinflammatory factors (interleukin-1α (Il-1α), Il-6, tumor necrosis factor-α (TNF-α), and transforming growth factor-β1 (TGF-β1), and matrix metalloproteinases (MMP2, MMP9, and MMP12). Indeed, quercetin effectively delayed pulmonary macrophage senescence after SiO_2_ exposure, thereby confirming the anti-silicosis and anti-aging effects.

Altogether, reports of this section highlight various endogenous and exogenous regulators of acute and chronic inflammation during health and diseases, and their potential clinical value.

